# Examining predictors of compassion fatigue among Iranian nurses: the role of personality traits and socio-emotional support

**DOI:** 10.1186/s12912-025-03604-w

**Published:** 2025-07-18

**Authors:** Marzieyh Malekiha, Leila Hosseinzadeh, Zeinab Zaremohzzabieh

**Affiliations:** 1 Department of Counseling, Hazrat-e Masoumeh University, Qom, Iran; 2 Islamic Azad University Khorasghan Branch, Isfahan, Iran; 3 Women and Family Studies Research Center, University of Religions and Denominations, Qom, Iran

**Keywords:** Compassion fatigue, Personality, Social support, Nurses, Cross-sectional studies, Iran

## Abstract

**Background:**

Compassion is a core element of helping professions, particularly nursing; however, prolonged exposure to emotional demands can lead to compassion fatigue, negatively impacting performance and care quality. This study aimed to predict the role of personality traits and socio-emotional support in compassion fatigue among nurses at Al-Zahra Hospital in Isfahan, Iran.

**Methods:**

A cross-sectional study was used to examine the predictors of compassion fatigue among nurses. The study sample consisted of 270 registered nurses employed at three public hospitals in Isfahan, Iran. A stratified sampling method was employed to ensure proportional representation from various hospital wards, including emergency, intensive care, and general units. Data were collected using a self-administered questionnaire that included demographics, the ProQOL for compassion fatigue, the NEO Personality Inventory, and the Socio-Emotional Support Scale (SESS). All statistical analyses were conducted using IBM SPSS Statistics for Windows, Version 26.0. Stepwise multiple linear regression identified predictors of compassion fatigue, and hierarchical regression tested interaction effects.

**Results:**

A stepwise multiple regression analysis revealed that extraversion (β = 0.531, *p* < .001), openness to experience (β = − 0.552, *p* < .001), neuroticism (β = 0.405, *p* < .001), and socio-emotional support (β = − 0.145, *p* = .011) significantly predicted compassion fatigue. Together, these variables explained 41.6% of the variance (*R²* = .416, *F*(4, 265) = 47.26, *p* < .001). A hierarchical regression further showed significant interaction effects: Neuroticism × Socio-emotional Support (*β* = 0.182, *p* = .004) and Openness × Socio-emotional Support (*β* = − 0.156, *p* = .015), increasing the total variance explained to 44.2% (*R²* = .442, *F*(6, 263) = 34.72, *p* < .001). These findings suggest that personality traits and socio-emotional support, including their interactions, play a significant role in predicting compassion fatigue among nurses.

**Conclusions:**

A significant portion of compassion fatigue among nurses was predicted by personality traits and socio-emotional support. These findings highlight the need for targeted interventions, such as psychological screening and support programs, tailored to individual risk profiles. Future qualitative research is recommended to explore nurses’ experiences with compassion fatigue in clinical settings.

## Introduction

Nursing is a profession that requires continuous emotional engagement and compassionate care, placing nurses at high risk for compassion fatigue. Compassion fatigue is defined as a state of emotional exhaustion resulting from prolonged exposure to the suffering of others, often leading to reduced empathy and emotional withdrawal [1, 2]. It comprises two key components: secondary traumatic stress, which results from exposure to others’ trauma, and burnout, which stems from prolonged workplace demands and emotional exhaustion [3–5]. In contrast, compassion satisfaction refers to the positive feelings derived from performing one’s caregiving role effectively, including fulfilment from helping others [3]. In nursing, the compassion fatigue is often used interchangeably with “cost of caring,” referring to the emotional exhaustion and psychological strain that emerge when supporting traumatized patients [6].

Nurses frequently care for patients experiencing intense pain, trauma, and distress. Over time, these encounters can diminish their emotional resilience and coping abilities [7, 8]. Although compassion fatigue shares similarities with burnout, the two differ in origin. Burnout typically arises from persistent organizational stress, while compassion fatigue stems from secondary exposure to patients’ trauma and emotional suffering [3, 4]. The symptoms of compassion fatigue are complex and may include anxiety, irritability, emotional numbness, depersonalization, and avoidance of patient interactions [8, 9]. These outcomes not only impair nurses’ psychological well-being but also negatively affect the quality and safety of patient care [2, 10]. As a result, compassion fatigue is recognized as a serious occupational health issue in the nursing profession.

The development of compassion fatigue can be understood through several theoretical frameworks. One particularly relevant model is the Conservation of Resources (COR) theory [11], which posits that individuals are motivated to retain, protect, and build resources such as emotional energy, social support, and psychological resilience [12]. When these resources are depleted through prolonged emotional labor without sufficient recovery, emotional exhaustion and psychological distress may follow. This framework helps explain individual variability in vulnerability to compassion fatigue among nurses [13]. In addition to environmental stressors, personality traits play a critical role in shaping this vulnerability. Costa and McCrae’s [14] Five Factor Model (FFM) identifies five major dimensions of personality—neuroticism, conscientiousness, agreeableness, extraversion, and openness to experience—which have been shown to influence emotional and behavioral responses to stress.

Neuroticism, defined by emotional instability, anxiety, and negative affect, has been consistently linked to increased susceptibility to compassion fatigue [15, 16]. Nurses high in neuroticism may be more likely to ruminate on distressing experiences and less able to manage emotional strain, resulting in faster emotional resource depletion [17]. In contrast, traits such as conscientiousness and agreeableness—typically associated with diligence, empathy, and social harmony—may paradoxically increase risk when nurses overextend themselves in caregiving roles without adequate self-care [18]. Extraversion presents a nuanced relationship with compassion fatigue. While extraverted nurses may initially form positive, empathetic patient relationships, their high sociability and emotional engagement can contribute to greater emotional exhaustion over time [19]. Finally, openness to experience, linked to creativity and cognitive flexibility, may offer a protective effect by enabling nurses to reframe and adapt to emotionally challenging situations [20, 21].

Beyond individual personality traits, environmental and social factors play a critical role in influencing vulnerability to compassion fatigue [22]. Social support theory, developed by Cohen and Wills [23], posits that perceived emotional support—feeling valued, understood, and connected to others—acts as a buffer against psychological stress. In nursing, socio-emotional support from colleagues, supervisors, and family members is essential for processing emotional experiences and recovering from work-related distress [24]. Emotional support, defined as the expression of empathy, trust, care, and reassurance, helps reduce the isolating effects of repeated exposure to patient trauma [25, 26]. Empirical studies have shown that nurses with strong emotional support networks are less likely to experience compassion fatigue, as these networks facilitate emotional regulation and the development of effective coping strategies [27, 28]. In contrast, nurses working in high-stress environments—such as intensive care or oncology—who lack sufficient emotional support are more susceptible to emotional exhaustion and secondary traumatic stress [29].

International research has emphasized the interplay of personality, empathy, and workplace support in shaping compassion fatigue. For example, Yu et al. [30] developed a predictive model of compassion fatigue among emergency nurses in China, identifying both personality traits and job conditions as significant predictors. Hunsaker et al. [31] found that job satisfaction and social support reduced compassion fatigue among emergency department nurses in the United States, while Nadarajan et al. [32] underscored the interaction between compassion satisfaction, burnout, and secondary trauma in Malaysian healthcare settings.

In the Iranian context, a growing body of evidence points to a high prevalence of compassion fatigue among nurses. Ariapooran [33] reported that 45.3% of Iranian nurses exhibited symptoms of compassion fatigue, while Salimi et al. [34] found that 42% were at high risk of burnout. Additional studies have shown that neuroticism significantly predicts emotional exhaustion [35], and that extraverted nurses may suffer emotional depletion when lacking peer support [36]. Kamali et al. [37] found that nurses with high levels of neuroticism and low perceived social support were more prone to health-related anxiety and emotional distress. However, few Iranian studies have comprehensively examined the combined impact of personality traits and socio-emotional support on compassion fatigue using theory-driven models such as the FFM and the COR.

Understanding the psychological and social predictors of compassion fatigue in Iran is essential for developing context-specific interventions that support nurses’ mental health and ensure the quality of patient care. Therefore, the present study seeks to examine the predictive roles of personality traits, based on the FFM, and socio-emotional support in determining compassion fatigue among Iranian nurses. Specifically, we hypothesize that: (1) neuroticism and low socio-emotional support will independently predict higher levels of compassion fatigue; (2) openness to experience and strong socio-emotional support will serve as protective factors; and (3) there will be significant interaction effects between personality traits and support levels. By integrating the COR theory with the FFM, this study provides a comprehensive framework to explore the antecedents of compassion fatigue in Iran’s healthcare setting. The findings are expected to guide evidence-based practices, such as personalized resilience training and enhanced support programs, aimed at reducing compassion fatigue and promoting sustainable nursing practice.

### Cultural and institutional context of Iranian public hospitals

Understanding compassion fatigue among Iranian nurses requires considering the broader cultural and institutional context of the country’s healthcare system. In Iran, the majority of healthcare services are delivered through public hospitals that are primarily funded by the government and partially supported by patients’ out-of-pocket payments and national insurance programs [38]. Despite national efforts to improve accessibility and equity, these hospitals often operate under significant resource constraints, such as outdated infrastructure, limited medical supplies, and insufficient budgets for workforce development [39]. The situation has been further exacerbated by ongoing economic sanctions and rising inflation, which have reduced hospitals’ purchasing power and undermined access to essential medications and equipment [40].

Staffing shortages remain a chronic issue in Iran’s public healthcare institutions. Nurse-to-patient ratios in many hospitals are significantly below global recommendations, particularly in critical units such as intensive care, emergency, and oncology wards [41]. A national-level assessment of nurse staffing patterns reported major deviations from the standards suggested by the Iranian Ministry of Health, leading to excessive workloads and reduced capacity for patient monitoring [42]. These structural imbalances contribute to high levels of job strain, emotional fatigue, and burnout among nursing staff.

Job satisfaction is another critical concern affecting both the stability and effectiveness of Iran’s nursing workforce. A systematic review by Amiresmaili and Moosazadeh [43] revealed that job satisfaction among Iranian nurses ranges between 46.3% and 51.9%, with variations depending on geographical region and institutional type. Specific sources of dissatisfaction include heavy workloads, inadequate compensation, insufficient staffing, outdated equipment, and assignment to non-nursing duties [44]. Although some studies report that human resource practices in public hospitals are slightly better than those in private ones, overall satisfaction remains alarmingly low, with some reports showing only 9.3% of nurses expressing high satisfaction levels [44]. On the other hand, factors such as a supportive workplace culture, spiritual meaning derived from caregiving, and strong internal motivation have been associated with improved job satisfaction and well-being [45].

To address these issues, scholars have recommended implementing flexible work arrangements, increasing investments in staffing and equipment, and enhancing opportunities for professional development and decision-making autonomy (Isfahani & Sarzehi, 2019). These institutional and cultural challenges form the backdrop against which compassion fatigue develops in Iran.

## Method

### Design

This study adopted a cross-sectional study. It was conducted and reported in accordance with the Strengthening the Reporting of Observational Studies in Epidemiology (STROBE) guidelines for observational research [46].

### Participants

The target population included all nurses employed at public hospital in Isfahan, Iran, during March 2024 (*N* = 877). To enhance the representativeness of the sample and reduce selection bias, a stratified random sampling method was adopted. Nurses were categorized into distinct strata based on their hospital departments (e.g., emergency, intensive care, pediatrics, and general wards). Participants were then selected proportionally and randomly from each department to ensure balanced representation across different clinical units and nursing specialties.

The required sample size was calculated using Krejcie and Morgan’s [47] sample size table, resulting in a target of 270 participants. Inclusion criteria were: (1) willingness to participate, (2) a minimum of five years of clinical work experience, and (3) no diagnosis of acute psychotic disorders. Nurses were excluded if they submitted incomplete questionnaires or were simultaneously enrolled in other formal training programs during the data collection period.

Data collection was carried out over a four-week period (1st March to 28th March, 2024). During this time, nurses were approached and surveyed across three public hospitals in Isfahan, with participation being entirely voluntary.

To provide context on participants’ professional qualifications, it is important to note that a Bachelor of Science in Nursing (BSN) in Iran typically requires four years of full-time academic study following secondary education. This degree includes both theoretical instruction and supervised clinical training. Nurses employed in specialized units such as intensive care, general wards, and pediatric departments are generally required to hold at least a bachelor’s degree. However, in some resource-limited hospitals, nurses with an associate degree (known as “Kardani”) may also be employed. These associate programs are typically two to three years in duration and offer more limited clinical exposure. Graduates from associate programs often face restricted eligibility for supervisory or advanced clinical roles compared to their BSN-qualified counterparts.

### Ethical considerations

This study received ethical approval from the Institutional Review Board (IRB) of Islamic Azad University, Khorasgan Branch, under the approval code IR.IAU.KHUISF.REC.1403.371. Following this approval and after obtaining permission from relevant authorities, the researchers visited public hospitals in Isfahan Province to recruit participants. Nurses who volunteered to participate were provided with detailed information about the study’s purpose, procedures, and their rights as participants, including the right to withdraw at any point without penalty. Questionnaires were distributed only to those who gave their informed consent. All participants signed an electronic informed consent form prior to completing the survey, and ethical principles of voluntary participation, confidentiality, and data protection were strictly observed throughout the research process. All methods were performed in accordance with the guidelines and regulations of the Declaration of Helsinki.

## Measures

### Demographic questionnaire

A researcher-developed questionnaire was used to collect comprehensive demographic data, including participants’ age (categorized into relevant ranges), gender (male, female), highest education level (e.g., high school, bachelor’s degree, postgraduate), and years of work experience (grouped by intervals). Additional details, such as employment status (full-time, part-time) and professional role, were also recorded to ensure a thorough understanding of the sample characteristics. This information provided essential context for analyzing potential associations between demographic factors and key study variables.

### Compassion fatigue questionnaire

The Professional Quality of Life Scale (ProQOL) developed by Stamm [48] consists of 30 items that assess three distinct subscales: compassion satisfaction, burnout, and secondary traumatic stress, with each subscale containing 10 items. Respondents are asked to rate how frequently they have experienced each statement over the past week using a 7-point Likert scale ranging from 0 (never) to 6 (very often). Example items include: *“I get satisfaction from being able to help people”* (compassion satisfaction), *“I feel worn out because of my work as a helper”* (burnout), and *“I jump or am startled by unexpected sounds”* (secondary traumatic stress). The Persian version of the ProQOL has been validated among Iranian healthcare providers, including nurses, showing strong reliability: *α* = 0.87 for compassion satisfaction, α = 0.87 for burnout, and *α* = 0.74 for secondary traumatic stress [49]. These findings confirm its cultural relevance and psychometric soundness in Iran. In the present study, the overall internal consistency of the scale was acceptable (*α* = 0.78). Subscale reliabilities were *α* = 0.86 for compassion satisfaction, *α* = 0.79 for burnout, and *α* = 0.82 for secondary traumatic stress.

### NEO five-factor inventory (NEO-FFI)

Personality traits were assessed using the 60-item NEO-FFI developed by Costa and McCrae [50], measuring five traits: Neuroticism, Extraversion, Openness, Agreeableness, and Conscientiousness. Items are rated on a 5-point Likert scale (0 = strongly agree to 4 = strongly disagree), with reverse scoring for specific items. The Persian version of the NEO-FFI has been culturally adapted and validated in Iranian samples. For example, Ghorbani and Montazer [51], and Sheykhshabani and Beshlideh [52] reported Cronbach’s alphas: *α* = 0.86 for Neuroticism, *α* = 0.73 for Extraversion, *α* = 0.56 for Openness, α = 0.68 for Agreeableness, and *α* = 0.87 for Conscientiousness, indicating acceptable internal consistency and construct validity. In the current study, Cronbach’s alphas were 0.84 (Neuroticism), 0.65 (Extraversion), 0.77 (Openness), 0.58 (Agreeableness), and 0.87 (Conscientiousness).

### Socio-emotional support (SESS)

Socio-emotional support was assessed using the 10-item *Socio-Emotional Support Scale* (SESS) [53], which evaluates two core dimensions: emotional sustenance (e.g., “*How often does someone listen to your worries?*”) and emotional validation (e.g., “*How often does someone make you feel valued?*”). Participants responded to each item using a 5-point Likert scale ranging from 1 (Never) to 5 (Always), with higher scores indicating greater perceived socio-emotional support. The original scale demonstrated strong internal consistency (*α* = 0.89; [53]. The Persian version, validated by Beyrami et al. [54], showed excellent psychometric properties among Iranian healthcare professionals, including high internal consistency (α = 0.91) and acceptable model fit indices (CFI = 0.93; RMSEA = 0.06).

### Statistical analysis

Data were analyzed using IBM SPSS Statistics (version 26.0; IBM Corp., Armonk, NY, USA) [55]. Descriptive statistics, including frequencies, percentages, means, and standard deviations, were computed to summarize demographic characteristics and key study variables. Initial data screening ensured normality, homogeneity, and the absence of outliers to meet statistical assumptions. Pearson’s correlation coefficients were calculated to examine bivariate relationships among variables, providing insight into potential associations before further analysis.

To explore predictive relationships, stepwise regression analysis was conducted to evaluate the effects of personality traits, social-emotional support, and demographic variables on compassion fatigue. This method allowed for the systematic inclusion of significant predictors while controlling for covariates. All statistical tests were two-tailed, with significance set at *p* < .05. Effect sizes were reported to assess practical significance, and multicollinearity diagnostics confirmed the independence of predictor variables. The robust analytical approach enhanced the reliability and validity of the findings, ensuring a comprehensive understanding of the factors influencing compassion fatigue.

### Validity and reliability

Content validity was ensured through expert consultation prior to data collection. A pilot study was conducted with 40 clinical nurses from one hospital to evaluate the reliability and clarity of the instruments. The Cronbach’s alpha coefficients for the main scales were all above 0.72, indicating acceptable internal consistency. Data were double-entered into SPSS by two independent researchers. Missing values and outliers were checked before conducting further analyses.

## Results

### Participant characteristics

The sample consisted of 270 nurses from Hospitals, Esfahan. Participants’ ages were distributed as follows: 46 (17%) aged 20–30 years, 77 (28.5%) aged 31–40 years, 116 (43%) aged 41–50 years, and 31 (11.5%) aged over 50 years. Education levels included 53 (19.6%) with an associate’s degree, 140 (51.9%) with a bachelor’s degree, and 77 (28.5%) with a master’s degree. Work experience was reported as follows: 37 (13.7%) with less than 5 years, 60 (22.2%) with 5–10 years, 39 (14.4%) with 11–15 years, and 134 (49.6%) with over 15 years.

### Descriptive statistics and normality

Descriptive statistics for personality traits, socio-emotional support, and compassion fatigue are presented in Table 1. The mean compassion fatigue score was 54.22 (*SD* = 10.88), indicating moderate levels. Personality traits showed varied distributions, with neuroticism (M = 25.69, SD = 6.06) and openness to experience (*M* = 24.01, *SD* = 4.57) being key predictors. Socio-emotional support had a mean of 45.06 (*SD* = 9.89).

The Kolmogorov-Smirnov test (Table 1) confirmed normality for most variables (*p* > .05), except neuroticism (*p* = .005) and openness (*p* = .013). However, regression analyses are robust to minor normality violations, and visual inspection of residual plots supported normality of errors.

**Table 1:** Descriptive statistics for study variables and Kolmogorov-Smirnov test for normality

Constructs	*M*	*SD*	MIN	MAX	*t*	*P*
EX	19.51	7.28	3	43	0.095	0.026
NE	25.69	6.06	9	42	0.110	0.005
CO	14.07	7.13	0	30	0.089	0.079
OE	24.01	4.57	13	34	0.102	0.013
AG	17.98	5.23	5	29	0.082	0.098
SE	45.06	9.89	14	60	0.086	0.102
CF	54.22	10.88	31	88	0.075	0.141

*Note* Extraversion = EX, Neuroticism = NE, Conscientiousness = CO, Openness to experience = OE, Agreeableness = AG, Socio-emotional support = SE, Compassion Fatigue CF

### Correlations

Pearson correlations (Table 2) revealed significant relationships between study variables and compassion fatigue. Neuroticism (*r* = .473, *p* < .001) was positively correlated. Openness to experience (*r* = −.199, *p* = .047) and socio-emotional support (*r* = −.373, *p* < .001) were negatively correlated. Unexpectedly, extraversion (*r* = .533, *p* < .001), conscientiousness (*r* = .461, *p* < .001), and agreeableness (*r* = .397, *p* < .001) also showed positive correlations.

**Table 2:** Correlation matrix for study variables

Construct	*r* with Compassion Fatigue	*P*
EX	0.533	< 0.001
NE	0.473	< 0.001
CO	0.461	< 0.001
OE	− 0.199	0.047
AG	0.397	< 0.001
SE	− 0.373	< 0.001

*Note* Extraversion = EX, Neuroticism = NE, Conscientiousness = CO, Openness to experience = OE, Agreeableness = AG, Socio-emotional support = SE

### Regression analyses

A stepwise multiple regression was conducted to test H_1_ and H_2_ (Tables 3, 4 and 5). In the final model (Step 4), extraversion (*β* = 0.531, *t* = 6.22, *p* < .001), openness to experience (*β* = -0.552, *t* = -5.02, *p* < .001), neuroticism (*β* = 0.405, *t* = 4.05, *p* < .001), and socio-emotional support (β = -0.145, *t* = -2.56, *p* = .011) significantly predicted compassion fatigue, explaining 41.6% of the variance (*R*^*2*^ = 0.416, F(4, 265) = 47.26, *p* < .001). The Durbin-Watson statistic (1.807) indicated independent errors. These results support H_1_ (neuroticism and low socio-emotional support as risk factors) and Hypothesis 2 (openness and socio-emotional support as protective factors).

**Table 3:** Stepwise regression model significance

Step	Predictors	SS	df	MS	F	*p*
1	EX	8782.35	1	8782.35	113.66	< 0.001
	Residual	20707.40	268	77.26		
2	EX, OE	10551.65	2	5275.82	74.38	< 0.001
	Residual	18938.11	267	70.92		
3	EX, OE, NE	11851.28	3	3950.42	59.57	< 0.001
	Residual	17638.47	266	66.31		
4	EX, OE, NE, SE	12278.72	4	3069.68	47.26	< 0.001
	Residual	17211.04	265	64.94		

*Note* Extraversion = EX, Neuroticism = NE, Conscientiousness = CO, Openness to experience = OE, Agreeableness = AG, Socio-emotional support = SE

**Table 4:** Stepwise regression model summary

Step	Predictors	*R*	*R*²	Durbin-Watson
1	EX	0.546	0.298	
2	EX, OE	0.598	0.358	
3	EX, OE, NE	0.634	0.402	
4	EX, OE, NE, SE	0.645	0.416	1.807

*Note* Extraversion = EX, Neuroticism = NE, Conscientiousness = CO, Openness to experience = OE, Agreeableness = AG, Socio-emotional support = SE

**Table 5:** Stepwise regression coefficients

Step	Predictor	B	SE	β	t	*p*	Tolerance	VIF
1	Constant	38.88	1.51		25.70	< 0.001		
	EX	0.774	0.073	0.546	10.66	< 0.001	1.000	1.00
2	Constant	52.01	3.00		17.32	< 0.001		
	EX	0.804	0.070	0.567	11.51	< 0.001	0.999	1.00
	OE	-0.573	0.115	− 0.246	-4.99	< 0.001	0.999	1.00
3	Constant	67.32	4.51		14.91	< 0.001		
	EX	0.595	0.082	0.420	7.23	< 0.001	0.871	1.15
	OE	-0.568	0.110	− 0.244	-5.12	< 0.001	0.998	1.00
	NE	0.443	0.100	0.256	4.42	< 0.001	0.871	1.15
4	Constant	73.75	5.12		14.39	< 0.001		
	EX	0.531	0.085	0.374	6.22	< 0.001	0.847	1.18
	OE	-0.552	0.110	− 0.237	-5.02	< 0.001	0.996	1.00
	NE	0.405	0.100	0.235	4.05	< 0.001	0.862	1.16
	SE	-0.145	0.057	− 0.135	-2.56	0.011	0.964	1.04

*Note* Extraversion = EX, Neuroticism = NE, Conscientiousness = CO, Openness to experience = OE, Agreeableness = AG, Socio-emotional support = SE

### Interaction effects

To test Hypothesis 3, a hierarchical regression examined interaction effects between personality traits (neuroticism, openness) and socio-emotional support. Variables were mean-centered to reduce multicollinearity. In Step 1, main effects were entered (Table 6). In Step 2, interaction terms (Neuroticism × Support, Openness × Support) were added. Results (Table 6) showed significant interactions: Neuroticism × Support (*β* = 0.182, *t* = 2.89, *p* = .004) and Openness × Support (*β* = -0.156, *t* = -2.45, *p* = .015). The model explained 44.2% of the variance (*R²* = 0.442, *F*(6, 263) = 34.72, *p* < .001). The positive Neuroticism × Support interaction indicates that high neuroticism combined with low support exacerbates compassion fatigue. The negative Openness × Support interaction suggests that high openness with high support further reduces compassion fatigue.

**Table 6:** Hierarchical regression for interaction effects

Step	Predictor	B	SE	β	t	*p*
1	Constant	73.75	5.12		14.39	< 0.001
	EX	0.531	0.085	0.374	6.22	< 0.001
	OE	-0.552	0.110	− 0.237	-5.02	< 0.001
	NE	0.405	0.100	0.235	4.05	< 0.001
	SE	-0.145	0.057	− 0.135	-2.56	0.011
2	Constant	73.80	5.08		14.52	< 0.001
	EX	0.520	0.084	0.366	6.19	< 0.001
	OE	-0.540	0.109	− 0.232	-4.95	< 0.001
	NE	0.390	0.099	0.226	3.94	< 0.001
	SE	-0.140	0.056	− 0.130	-2.50	0.013
	NE × SE	0.012	0.004	0.182	2.89	0.004
	OE × SE	-0.010	0.004	− 0.156	-2.45	0.015

## Discussion

This study investigated the predictors of compassion fatigue among nurses working in public hospitals in Isfahan Province, employing the COR theory [11, 56] and FFM of personality [57]. Specifically, it explored how neuroticism, openness to experience, and extraversion—as well as socio-emotional support—predict compassion fatigue and how these factors interact. The findings largely supported the study’s hypotheses and provided meaningful theoretical and practical insights. The results emphasize the complex interplay between individual personality traits and contextual resources in determining emotional outcomes in high-stress healthcare environments [58, 59].

In line with Hypothesis 1, neuroticism was positively associated with compassion fatigue, supporting previous research and theoretical expectations. Nurses high in neuroticism, who typically exhibit emotional instability, anxiety, and self-doubt, may struggle to regulate stress and are more vulnerable to emotional exhaustion [60]. This is consistent with COR theory’s view that individuals with poor emotional regulation possess fewer internal resources to cope with stressors, making them more susceptible to compassion fatigue [61]. Conversely, openness to experience significantly and negatively predicted compassion fatigue, suggesting that individuals who are more imaginative, flexible, and cognitively open may reframe stressful situations more adaptively, thereby conserving emotional resources [62]. This supports both the COR model and Figley’s [58] conceptualization of openness as a protective factor in caregiving professions.

Interestingly, extraversion also emerged as a significant positive predictor of compassion fatigue, though it was not initially hypothesized. While extraversion is commonly associated with resilience, sociability, and positive affect, in the emotionally intense field of nursing, it may increase the likelihood of emotional over-engagement [63]. Extraverted nurses may invest more energy in interactions with patients and co-workers, leading to greater emotional labor and quicker resource depletion—aligning with COR theory’s assertion that frequent emotional demands accelerate burnout [56, 64, 65]. On the other hand, conscientiousness and agreeableness did not show significant effects, suggesting that while these traits may support work ethic and teamwork, they do not directly buffer emotional exhaustion unless paired with appropriate coping strategies or external support [66].

Socio-emotional support emerged as a significant protective factor, confirming H_2_. Support from family and significant others—more so than friends—was associated with lower levels of compassion fatigue, likely because of its consistent and emotionally validating nature [67]. According to COR theory, such support replenishes depleted emotional reserves and serves as an external buffer against the strain of caregiving [61]. This finding also echoes cognitive coping theory, which posits that emotionally expressive support facilitates the processing and regulation of stress [68]. Nurses who receive stable, affirming support are thus more likely to manage their emotional responses effectively and maintain psychological resilience [62].

Finally, the study’s findings on interaction effects reinforced the importance of resource-context congruence, in line with H_3_. The interaction between neuroticism and support revealed that nurses with high neuroticism are particularly vulnerable to compassion fatigue in the absence of socio-emotional support [60]. Conversely, those high in openness benefited the most when support was also high, highlighting a buffering synergy between internal traits and external resources [62]. These results not only refine the COR model by illustrating the moderating role of support in the personality-fatigue link but also underscore the need for personalized intervention strategies. For example, support programs tailored to nurses’ personality profiles—such as resilience training for neurotic individuals and reflective practices for open individuals—can enhance emotional well-being and reduce compassion fatigue [31]. Healthcare institutions should thus move beyond generic burnout prevention models and foster individualized support systems that account for both personality differences and social resource availability.

### Implications

#### Theoretical implications

This study makes four key theoretical contributions to the literature. First, it extends the COR theory [56, 61] by empirically demonstrating how internal resources, such as personality traits (e.g., neuroticism, openness), and external resources, such as socio-emotional support, jointly influence compassion fatigue among nurses. While COR theory has been widely applied to stress and burnout, few studies have integrated both individual dispositions and contextual support systems within a single model in healthcare settings. Second, this research advances the FFM of personality by confirming the expected roles of neuroticism and openness and revealing an unexpected positive association between extraversion and compassion fatigue.

The finding challenges traditional views of extraversion as purely protective and suggests that in emotionally demanding professions like nursing, extraversion may increase vulnerability through heightened emotional engagement and labor. Third, the study identifies socio-emotional support as a key moderator, refining the COR framework by showing how the interaction between personal traits and social resources influences emotional outcomes, consistent with calls for more dynamic models of stress and resilience [11]. Finally, by focusing on Iranian nurses in public hospitals, the study addresses a gap in the compassion fatigue literature in non-Western contexts and contributes to the cross-cultural validation of both COR theory and the FFM, enhancing their relevance and applicability in diverse healthcare environments.

#### Implications for nursing practice

The findings of this study have several practical implications for nursing practice, particularly in emotionally demanding healthcare environments. First, recognizing the role of personality traits such as neuroticism and openness can help nursing administrators tailor emotional resilience programs to individual needs. For instance, nurses high in neuroticism may benefit from cognitive-behavioral interventions aimed at improving emotional regulation, while those high in openness may respond well to reflective and creative coping strategies. Second, the identification of socio-emotional support as a significant protective factor against compassion fatigue underscores the need for healthcare institutions to foster supportive work environments. This can include formal peer support systems, regular debriefing sessions, and family-inclusive wellness programs.

Third, given the high emotional burden inherent in trauma-related care, especially in pediatric and critical care settings, structured educational and psychosocial interventions are essential. Research shows that nurses benefit from simulation-based training, structured debriefings, and compassion fatigue workshops that increase awareness of trauma dynamics and teach effective coping strategies [6, 69]. These interventions help prevent emotional detachment and promote emotional resilience, spiritual well-being, and long-term professional identity development.

Moreover, the emotional responses of nurses to trauma and patient loss should be seen not only as psychological phenomena but also as social processes embedded in the work culture of each unit. Normative organizational expectations often shape how emotions are expressed or suppressed in clinical settings. Therefore, educational programs must also address team norms and emotional climates, helping nurses collectively develop healthy emotional coping mechanisms within their professional communities [6].

Finally, the unexpected positive relationship between extraversion and compassion fatigue suggests that even typically resilient personality types may be at risk due to over-engagement. This highlights the importance of emotional labor training and workload management policies that prevent excessive emotional strain, even among seemingly well-adjusted nurses.

## Conclusion

This study highlights the critical role of both individual personality traits and external socio-emotional support in shaping compassion fatigue among nurses. Specifically, neuroticism and extraversion emerged as key risk factors, while openness to experience and socio-emotional support functioned as protective factors. These findings reinforce the theoretical value of integrating the COR theory and the FFM in explaining emotional exhaustion in healthcare settings. In light of these results, it is recommended that healthcare institutions implement resilience-building interventions tailored to specific personality profiles—such as emotional regulation training for neurotic individuals and reflective practices for open individuals. Additionally, because compassion fatigue is influenced by the emotional norms and implicit cultures within clinical units, systemic strategies should also aim to cultivate emotionally supportive team environments. Therefore, mitigating compassion fatigue requires a dual approach: strengthening individual coping resources and fostering unit-level emotional climates that support long-term psychological resilience and professional sustainability in nursing.

### Limitations and future directions

Despite its contributions, this study is not without limitations. First, the cross-sectional design restricts causal inferences. Future research should build on these findings by employing longitudinal designs to explore how compassion fatigue evolves over time in response to fluctuations in personality-related coping and changes in workplace support structures. Second, the use of self-reported questionnaires may have introduced bias; incorporating supervisor assessments or peer evaluations in future work would enhance validity and provide a fuller picture of emotional exhaustion in clinical settings.

Third, this study focused exclusively on nurses working in public hospitals in Isfahan Province. Future research could replicate this model in other regions or private hospitals to examine whether the relationships between neuroticism, extraversion, openness, and support hold across different healthcare contexts. For example, the influence of workplace emotional culture may vary in high-resource versus low-resource settings. Fourth, since this study identified extraversion as a surprising risk factor, further qualitative work could investigate why and how socially engaged nurses may experience emotional overextension, possibly due to implicit emotional norms in specific units like ICUs or pediatric wards.

Fifth, this study revealed that compassion fatigue is shaped not only by individual traits but also by unit-level emotional expectations. Future research should directly explore these emotional norms—through ethnographic studies or unit climate surveys—to better understand how local culture moderates the trait-fatigue relationship. Finally, while we tested moderating effects of socio-emotional support, future models might include emotional labor or organizational justice as mediators, offering insight into the psychological mechanisms linking personality, support, and fatigue in high-stress caregiving roles.

## Data Availability

Data will be provided upon reasonable request.

## Abbreviations

COR: Conservation of Resources
FFM: Five Factor Model
NEO-FFI: NEO Five-Factor Inventory
ProQOL: Professional Quality of Life Scale
SESS: Socio-Emotional Support
STROBE: Strengthening the Reporting of Observational Studies in Epidemiology
